# Evaluating Mobile LiDAR Intensity Data for Inventorying Durable Tape Pavement Markings

**DOI:** 10.3390/s24206694

**Published:** 2024-10-17

**Authors:** Gregory L. Brinster, Mona Hodaei, Aser M. Eissa, Zach DeLoach, Joseph E. Bruno, Ayman Habib, Darcy M. Bullock

**Affiliations:** 1 Joint Transportation Research Program, Lyles School of Civil and Construction Engineering, College of Engineering, Purdue University, West Lafayette, IN 47907, USA; mhodaei@purdue.edu (M.H.); eissaa@purdue.edu (A.M.E.); ahabib@purdue.edu (A.H.); darcy@purdue.edu (D.M.B.); 2 Indiana Department of Transportation, 100 N Senate Ave, Marion County, Indianapolis, IN 46204, USA; zdeloach@indot.in.gov (Z.D.); jbruno@indot.in.gov (J.E.B.)

**Keywords:** LiDAR, pavement markings, preformed tape

## Abstract

Good visibility of lane markings is important for all road users, particularly autonomous vehicles. In general, nighttime retroreflectivity is one of the most challenging marking visibility characteristics for agencies to monitor and maintain, particularly in cold weather climates where agency snowplows remove retroreflective material during winter operations. Traditional surface-applied paint and glass beads typically only last one season in cold weather climates with routine snowplow activity. Recently, transportation agencies in cold weather climates have begun deploying improved recessed, durable pavement markings that can last several years and have very high retroreflective properties. Several dozen installations may occur in a state in any calendar year, presenting a challenge for states that need to program annual repainting of traditional waterborne paint lines, but not paint over the much more costly durable markings. This study reports on the utilization of mobile mapping LiDAR systems to classify and evaluate pavement markings along a 73-mile section of westbound I-74 in Indiana. LiDAR intensity data can be used to classify pavement markings as either tape or non-tape and then identify areas of tape markings that need maintenance. RGB images collected during LiDAR intensity data collection were used to validate the LiDAR classification. These techniques can be used by agencies to develop accurate pavement marking inventories to ensure that only painted lines (or segments with missing tape) are repainted during annual maintenance. Repeated tests can also track the marking intensity over time, allowing agencies to better understand material lifecycles.

## 1. Introduction

Pavement markings are essential for delineating travel lanes. Historically, this has been done with paint containing some type of nighttime retroreflective material embedded in the binder. There is considerable innovation underway across the country regarding pavement markings, particularly in improving nighttime visibility at a cost-effective price. As a result, agencies are diversifying their portfolios of pavement marking materials and application methods. These markings are typically installed as part of pavement resurfacing projects over several years and are geographically distributed across large regions.

Table 1a summarizes the installation cost (per mile) of emerging durable marking materials: thermoplastic, epoxy, and preformed tape. Table 1b summarizes the approximate number of lane miles of these materials installed along state-maintained roads in Indiana as of May 2024. Thermoplastic, epoxy, and preformed tape markings all contain retroreflective elements that are designed to reflect the light emitted from a vehicle’s headlights back to the driver, to provide good nighttime visibility of pavement markings. The depth at which the beads are installed in the marking is a factor of air temperature, material temperature, and bead size. Any fluctuations in these variables while applying thermoplastic and epoxy markings may cause the beads to either be too shallow or deeply embedded in the marking material. In these cases, the glass beads do not have the same retroreflective properties and scatter the incoming light rather than reflecting it back to the driver. Preformed tape, however, is made in a controlled environment, which allows the companies to monitor and control the glass bead embedment. This results in increased material cost, but also a significant improvement in retroreflectivity consistency.

According to the 2023 Manual on Uniform Traffic Control Devices (MUTCD), Section 3A.05 [1], the minimum retroreflectivity for roadways with speed limits of 35 mph and greater is 50 $mcd/m^{2}/lx$, while roadways with speed limits of 70 mph or greater have a minimum recommended level of 100 $mcd/m^{2}/lx$. INDOT’s minimum initial retroreflectivity values for paint, thermoplastic, epoxy, and preformed tape can be found in Table 2. These minimum values are paired with the retained values for 1 and 2 years after installation. Although the minimum values for thermoplastic and epoxy are greater than or equal to 300 $\;mcd/m^{2}/lx$, it is common to see retroreflectivity values over 500 $\;mcd/m^{2}/lx$ for well-installed markings. Preformed tape also commonly has a greater retroreflectivity value than the minimum of 650 $\;mcd/m^{2}/lx$.

### Motivation

**Figure 1:**
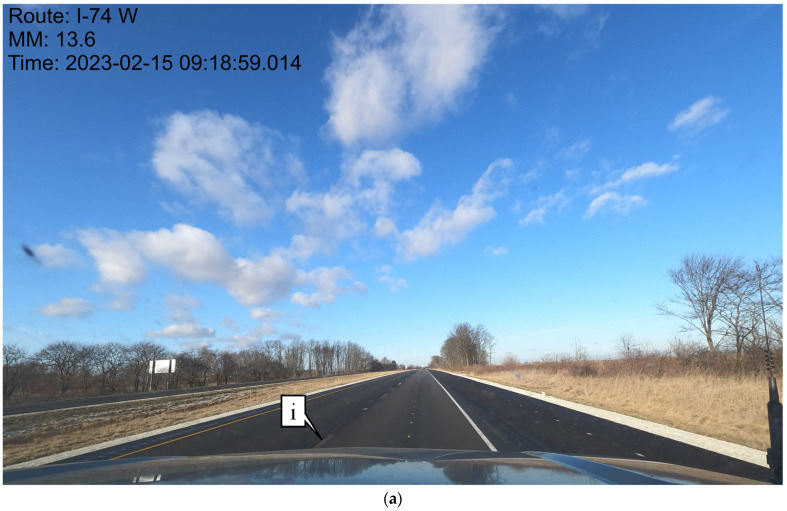

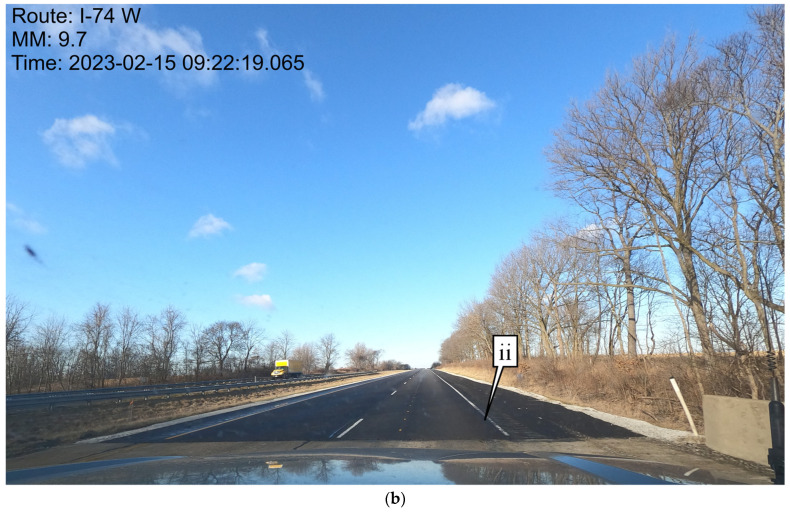
Examples of durable tape markings on I-74 West that need maintenance. (**a**) Skip lines; (**b**) edge line.

Although construction records can be used to develop geospatial marking inventories, the process is very labor-intensive and prone to error, particularly when construction projects encounter unplanned conditions and/or change orders. For example, during a 2023 resurfacing project, preformed durable tape markings were applied to a section of I-74 in Indiana between mile markers 0 and 16. Unfortunately, some of the markings along the 16-mile segment did not adhere properly to the new pavement and left behind the recessed groove with no marking. Examples of missing skip lines can be seen in Figure 1a, highlighted by callout i, and missing edge lines can be seen in Figure 1b, highlighted by callout ii. When these situations occur, it is important to map the impacted areas and develop a remediation plan. Although traditional manual mapping can be performed, developing an automated procedure to identify the impacted area provides a more systematic approach with less worker exposure. 

In this situation, the missing durable tape markings were replaced with waterborne painted lines. As a result, it was particularly important to have a high-fidelity map of where paint and tape were applied. This also provides an ideal test bed for evaluating mobile mapping technology that could not only map the locations of lines but also infer the type of material used for the lines. In addition, the presence of sections of recessed preformed tape markings that met agency standards, recessed preformed tape markings that did not meet agency standards, and traditional waterborne paint markings make this section ideal. The following sections detail the different types of pavement markings, their approximate installed cost, the importance of identifying precise locations of pavement marking materials (tape, thermoplastic, paint), and mobile mapping technology. The paper concludes with a series of case studies demonstrating how mobile mapping technology can be used to produce a high-resolution map of pavement marking types.

## 2. Materials and Methods

### 2.1. Material Types and Test Site Characteristics

**Figure 2:**
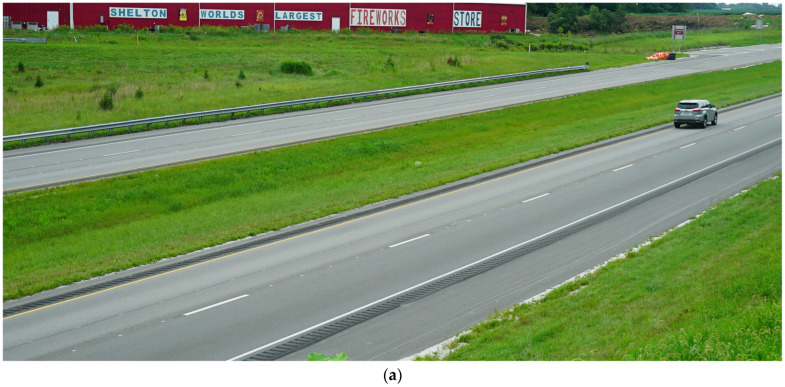

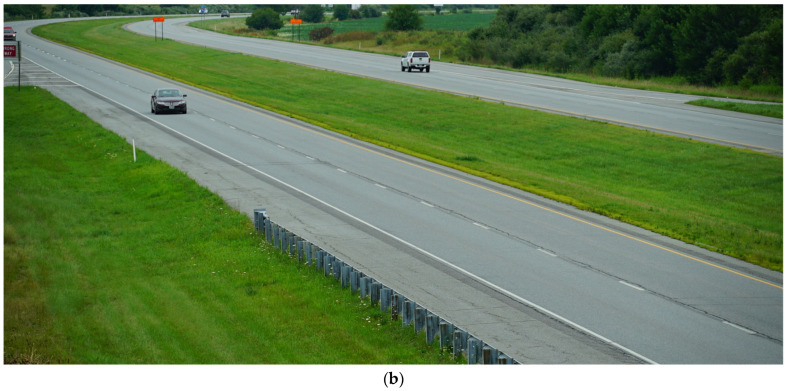
Lane markings on I-74. (**a**) Durable tape; (**b**) waterborne paint.

Figure 2 shows overview images of two different sections of I-74. Figure 2a is a section containing the recessed durable preformed tape markings and Figure 2b is one with waterborne paint markings. It is very difficult to discern the type of pavement marking in each of the photos and even harder while driving on the road at highway speed. 

**Figure 3:**
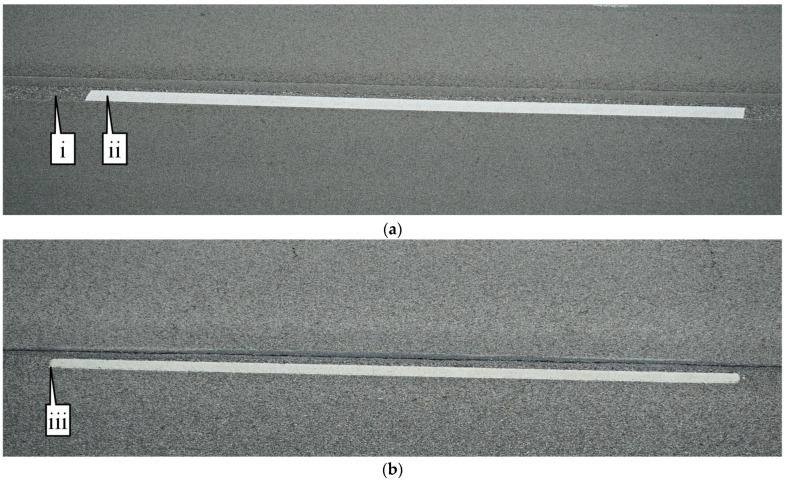
Close-up images of lane markings on I-74 (images taken along bridge deck shoulders using a DSLR Camera). (**a**) Recessed durable tape; (**b**) waterborne paint.

Figure 3 shows a zoomed-in view of the two types of marking; Figure 3a is a photo of recessed durable preformed tape and Figure 3b is a photo of waterborne paint. Even when zoomed in close to the markings, it is difficult to identify the marking type unless looked at with a well-trained eye. Callout I points to the recessed section of the marking. This recession is milled into the asphalt and allows the marking to sit below the top surface of the pavement, protecting it from snowplows and excessive tire wear. Callout ii points to the beginning of the marking in the recessed groove. The waterborne paint marking in Figure 3b is observed to have some increased degradation near callout iii. This marking is in the eastbound direction, with vehicles traveling from left to right across the image.

### 2.2. Literature Review: LiDAR Intensity as a Screening Tool for Retroreflectivity

LiDAR-based mobile mapping is an emerging technology that has been adopted by multiple public and private agencies for mapping and assessing roadway assets such as pavement markings. Compared to the traditional method of handheld and, recently, mobile retroreflectometers, LiDAR systems have the ability to simultaneously collect data across multiple lanes. Mobile retroreflectometers can only collect data from the lane markings immediately on either side of the mapping vehicle, requiring agencies to perform drives along each individual lane of the test corridor. LiDAR also has the ability to collect data for a variety of other applications, such as ditch line mapping [2], bridge deck analysis [3], pavement quality assessment [4,5], pothole mapping [6], signage visibility [7], etc. The diversity of data applications makes LiDAR more cost-effective due to the ability to spread the costs across multiple projects.

**Figure 4:**
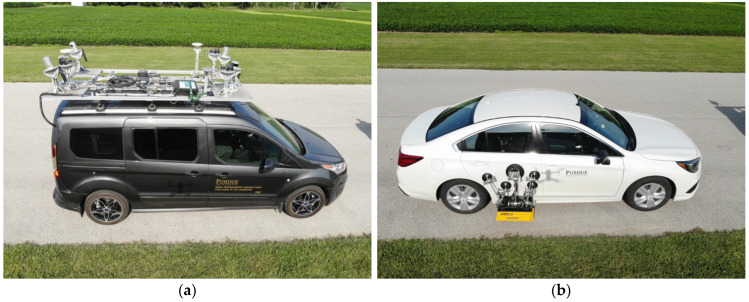
Vehicles used for data collection in 2021. (**a**) Mobile LiDAR-mapping Ford Transit; (**b**) mobile retroreflectometer.

**Figure 5:**
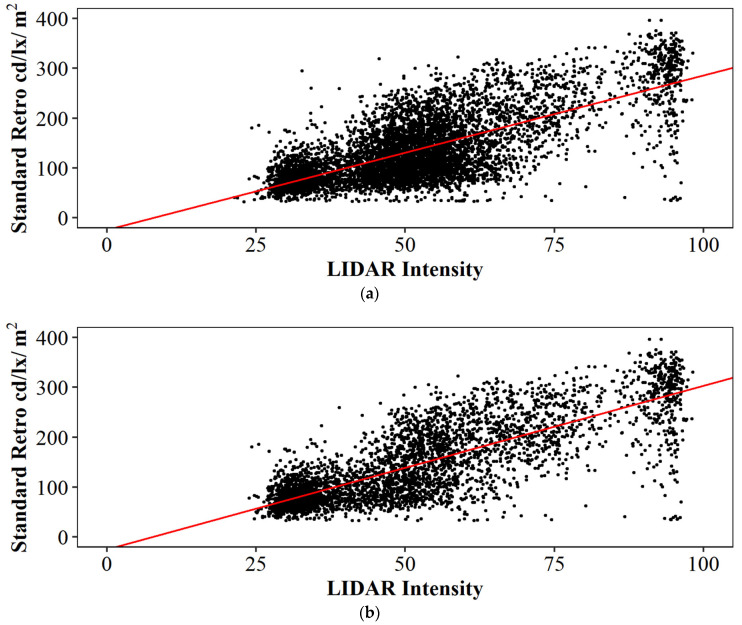
Center skip line linear correlation of 2021 LiDAR intensity and standard retroreflectivity (adapted from [27]). (**a**) US-52 and US-41 combined; (**b**) US-41.

Current mobile mapping technologies can extract and evaluate pavement markings [8,9,10,11,12,13,14,15,16,17,18,19,20,21,22,23,24,25,26]. Past research [27] has demonstrated a very good correlation between LiDAR intensity and retroreflectivity. The collection reported on in this study [27] was completed using a LiDAR-equipped Ford Transit, as seen in Figure 4a. This vehicle [28] is equipped with three Velodyne HDL-32e [29] sensors and one VLP-16 [30] LiDAR sensor. There are also three RGB cameras attached to the vehicle and a global navigation satellite system/inertial navigation system (GNSS/INS) unit with supporting antennas [31]. Retroreflectivity data were collected by a passenger car that was equipped with a Road Vista Laserlux G7 (LLG7) [32], as seen in Figure 4b. During that research, approximately 70 lane miles of mobile mapping LiDAR intensity [33] was collected concurrently with retroreflectivity. Figure 5 shows a quantitative correlation between LiDAR intensity and standard retroreflectivity data collected in a previous study [27].

### 2.3. Data Collection Techniques

**Figure 6:**
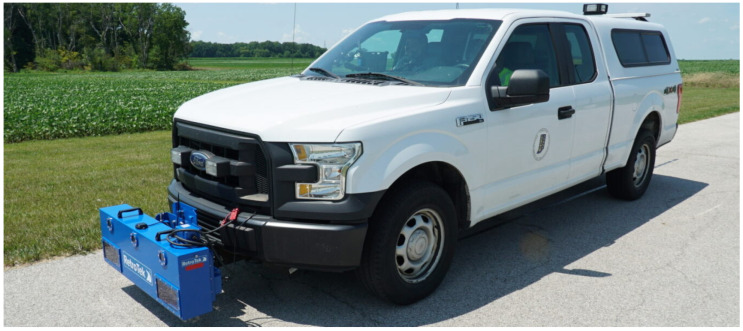
INDOT fleet vehicle with RetroTek-D unit attached.

Based on these promising results (Figure 5), several follow-up data collections were conducted. Intensity data were collected using the same LiDAR-equipped Ford Transit mentioned earlier and pictured in Figure 4a. The HDL-32e sensors are capable of capturing around 700,000 points per second at a maximum range of 100 m. The VLP-16 captures 300,000 points per second at a maximum range of 100 m. These sensors operate at an accuracy of ±2 and ±3 cm, respectively [28]. Retroreflectivity data were collected using a RetroTek-D [34] unit; Figure 6 shows this device attached to an INDOT fleet vehicle. This system has a measurement frequency of 1000 lines per second and a retroreflectivity accuracy of ±5%.

### 2.4. Qualitative Comparison of Pavement Markings’ Nighttime Visibility and LiDAR Intensity

**Figure 7:**
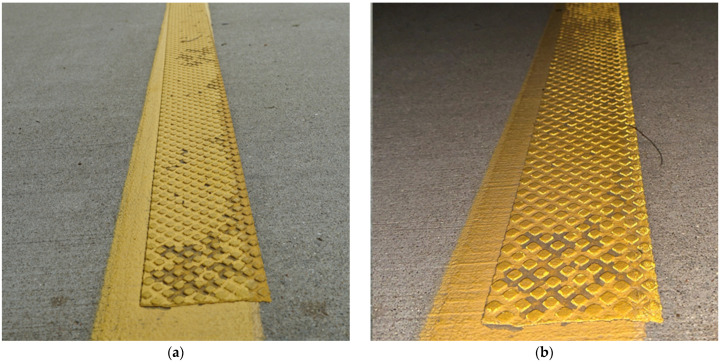

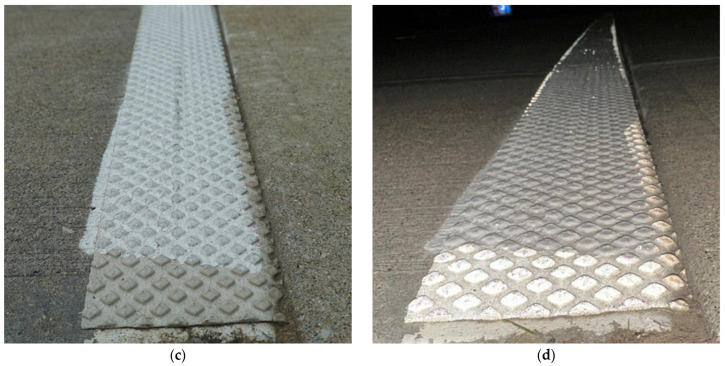
Comparison of painted-over tape with and without camera flash. (**a**) Yellow without flash; (**b**) yellow with flash; (**c**) white without flash; (**d**) white with flash.

As states are starting to encounter a heterogeneous environment of pavement markings, it is important to ensure the paint is not applied over durable markings, particularly tape. Once painted over, the preformed tape loses nearly all of its nighttime reflectiveness. To better visualize the correlation between nighttime visibility and LiDAR intensity, a small-scale study was completed. Figure 7 shows multiple preformed tape markings along a bike path in West Lafayette, IN, that have been partially painted over, causing a substantial reduction in retroreflectivity. In Figure 7a,c, the images were taken without the camera flash, resulting in a relatively dull marking. Figure 7b,d were taken with the camera flash turned on, highlighting the edges of the preformed tape that had not been painted over. 

**Figure 8:**
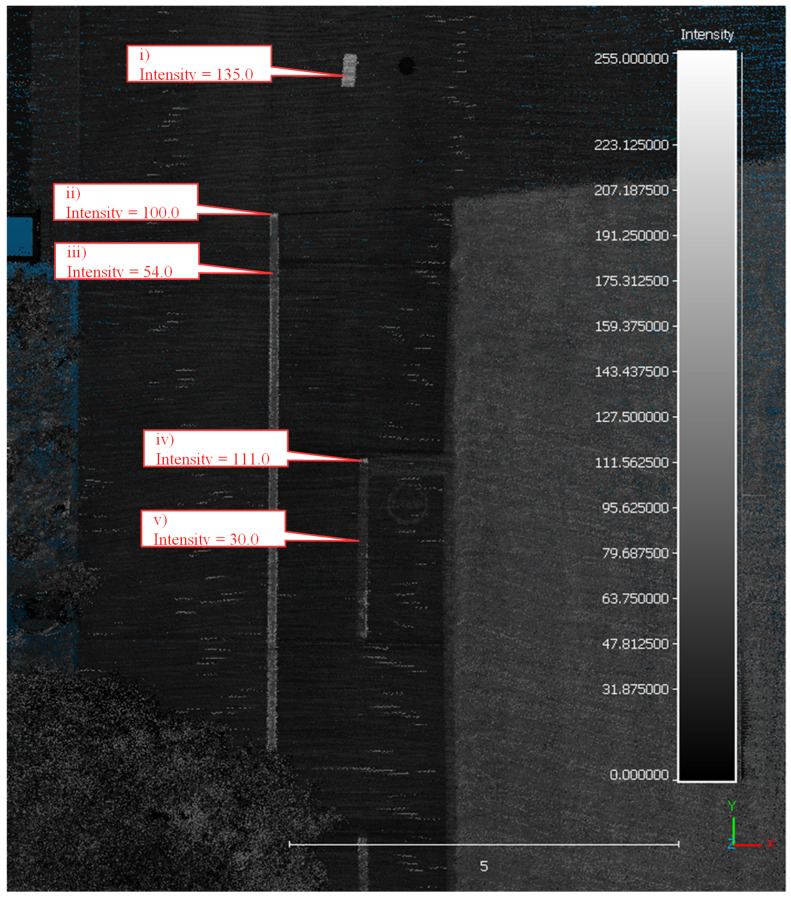
Top-down view of LiDAR-generated point cloud with callouts to 5 key locations.

**Figure 9:**
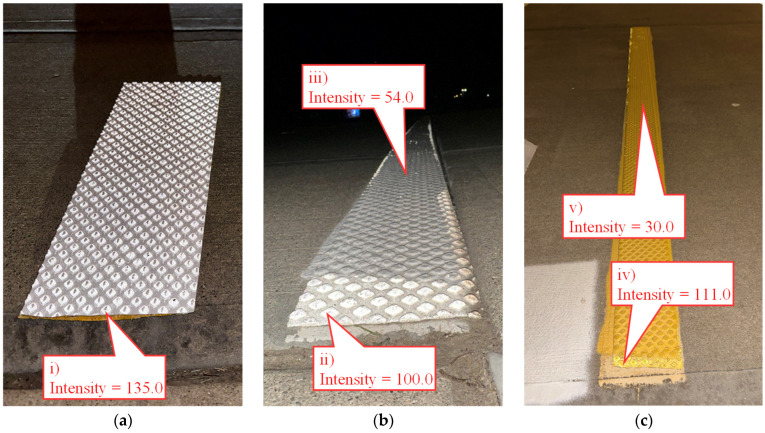
Close-up view of the 5 key features. (**a**) Sample section; (**b**) painted-over white tape (installed in 2018); (**c**) painted-over yellow tape (installed in 2018).

This change in retroreflectivity is quantified in Figure 8, where a mobile LiDAR system was deployed to collect data along the bike path. The intensity values recorded by the mobile LiDAR system are in the 0 to 255 range. Callout i points to a sample piece of preformed tape and acts as a control for the data collection. This piece is brand new and represents a newly installed segment of white preformed tape. The LiDAR intensity value corresponding to the control segment is 135. Callout ii points to the end of a white preformed tape edge line that had been painted over. This location, however, was not painted over and still has a very high intensity value of 100, 6 years after installation (2018–2024). Slightly further down the marking, callout iii points to a segment of the same edge line that had been painted over. This significantly impacts the intensity of the marking, reducing it by nearly half to a value of 54. The same pattern holds true for the yellow center line, callout iv points to an unpainted section with an intensity of 111, and callout v points to a painted-over section with an intensity of 30. Close-up RGB images of these same areas of interest can be seen in Figure 9.

## 3. Results

**Figure 10:**
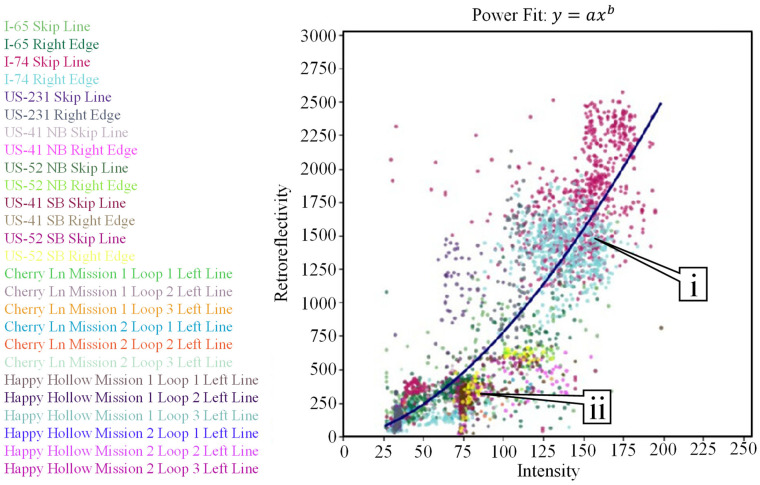
Power regression of retroreflectivity and intensity for 26 data sets across 528 miles.

**Figure 11:**
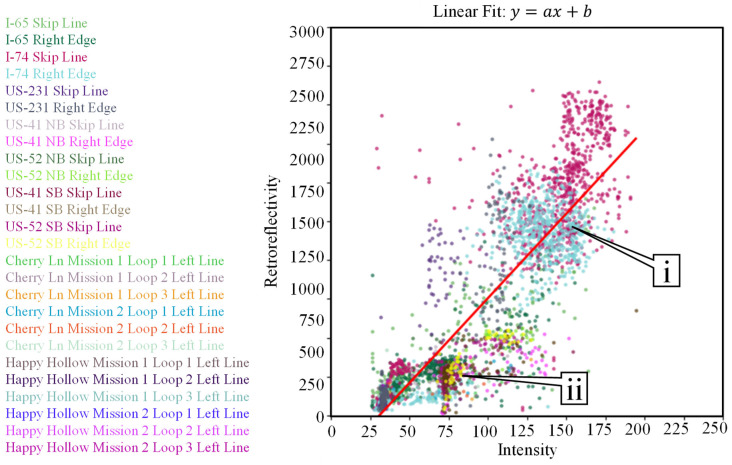
Linear regression of retroreflectivity and intensity for 26 data sets across 528 miles.

A total of 26 individual data sets were extracted from three data collections across 528 lane miles and analyzed. Each of these data collections was conducted along dry roads in the absence of precipitation. PCC (Pearson Correlation Coefficient (r)) is used to measure the linear relationship between retroreflectivity and LiDAR intensity, while the CD (Coefficient of Determination ($R^{2}$)) represents the model’s goodness of fit. Figure 10 shows the data plotted with a power fit line, with a PCC of r = 0.86 and a CD of $R^{2}=0.76$. Figure 11 shows the data plotted with a linear fit line, with a PCC of r = 0.86 and a CD of $R^{2}=0.74$. A Summary of the different parameters can be found in Table 3. For the power fit mode, the “a” term represents the coefficient and the “b” term represents the exponent. In the linear model, “a” represents the slope of the fit line while “b” represents the y-intercept. The RSME (Root Mean Square Error) is calculated by adding the mean value to the standard deviation for each of the two models. This value represents the average difference between the estimated value and the actual value. In this case, because retroreflectivity is the dependent variable, this RMSE value represents a retroreflectivity amount, measured in $mcd/m^{2}/lx$.

Power and linear models were selected for this analysis due to their large correlation values and simplicity. Other models, such as exponential and logarithmic functions, were examined but did not provide as well of a representation compared to the power and linear fit models.

Compared to previous work in this space [8], the power exponent in this study is slightly larger and greater than 1. This difference can be explained by the addition of recessed, durable preformed tape markings. These markings have much greater retroreflectivity and intensity values, significantly expanding the data range. Figure 10, callout i, points to the location of these markings in the plot. Both studies have a similar $R^{2}$ value ([8], Figure 3 $R^{2}=0.7996$) and this study reports $R^{2}=0.76$ (Figure 10 and Table 3).

Looking at the regression plots, some collections cluster tighter than others, based on the materials present in the corridors. Preformed tape markings tend to have more stochastic variation, but larger overall, intensity and retroreflectivity values compared to waterborne paint and even durable thermoplastic and epoxy. Callout i points to the tape marking clusters while callout ii points to the non-tape marking clusters.

Although both trends are very strong, it is also clear there is substantial stochastic variation in the data. This paper takes a detailed look at two of the data collection runs (I-74 collected on 18 September 2023, and the West Lafayette Test Loop collected on 9 April 2024) and uses dashcam images to explain some of the apparent outliers. After examining some of these outliers, we assessed that the fidelity of the LiDAR intensity data can help agencies derive important insights into their pavement markings.

### 3.1. I-74 Case Study

#### 3.1.1. Methods

**Figure 12:**
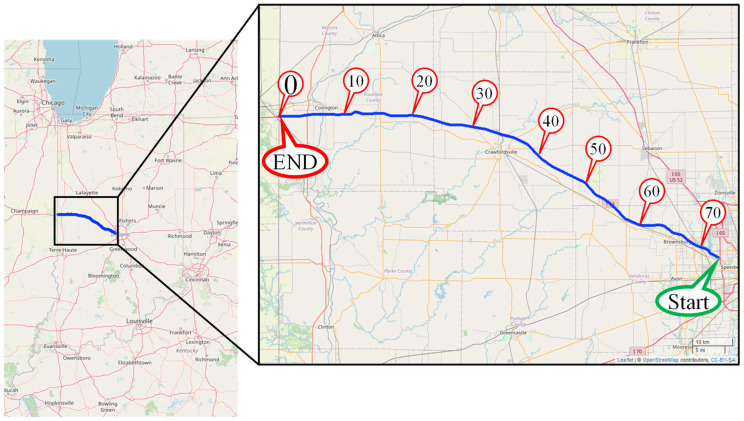
Map of 73-mile test segment along I-74 in Western Indiana.

During a scouting mission in early 2023, it was discovered that some of the late season 2022 preformed tape markings along I-74 had failed and the recessed grooves where they had been applied did not contain any markings. These areas with missing tape were repaired by applying paint. This heterogeneous set of markings served as an ideal test site for mobile LiDAR mapping data collection, which was completed in September 2023. This data collection was conducted along a 73-mile stretch of westbound I-74 in Indiana. The section of I-74 is located just west of Indianapolis, stretching from the I-465 interchange in Speedway, IN, to the Illinois state border near Covington, IN. Figure 12 shows the location of the test route and direction of travel.

**Figure 13:**
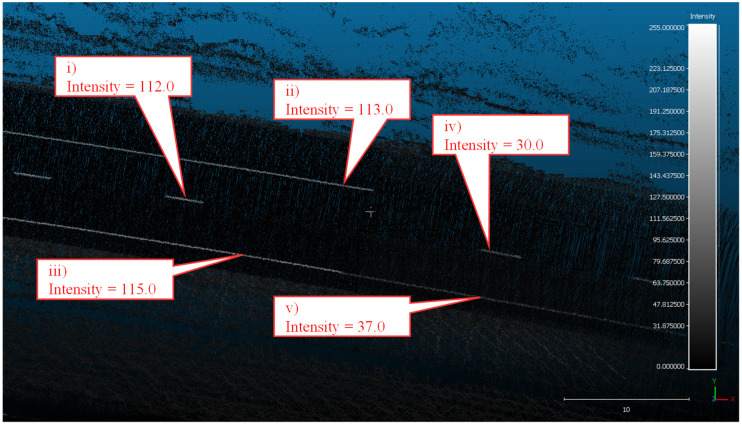
Top-down view of LiDAR-generated point cloud along I-74 with callouts to 5 key locations.

For this section of westbound I-74, over 1.3 million lane-marking data points were extracted from the point cloud. A sample point cloud can be visualized for a section of I-74 near MM 16 in Figure 13. This specific section of I-74 is the transition from waterborne paint to the preformed tape, seen by the increase in intensity of callouts i, ii, and iii compared to iv and v.

**Figure 14:**
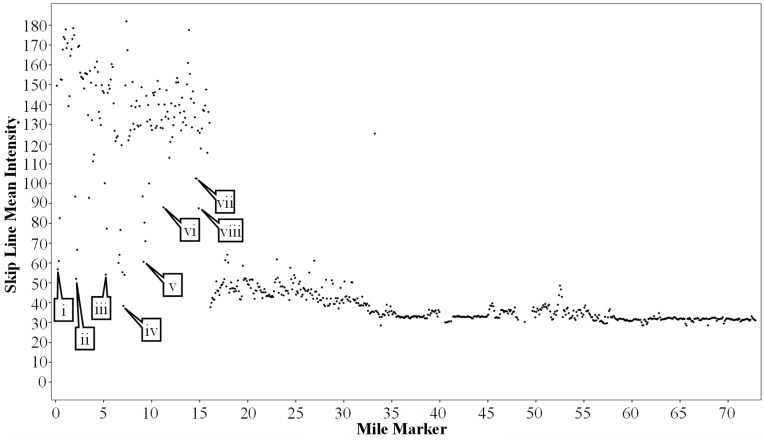
I-74 LiDAR intensity for skip lines over the 73-mile test segment with callouts to AOIs (Areas of Interest).

Once the intensity values are extracted and parsed into skip line, left and right edge lines, they are linearly referenced from XYZ coordinates to the interstate mile marker values to get a relative spatial awareness. These data are then plotted, with linear mile markers as the independent variable and intensity as the dependent variable. Figure 14 shows the data from the westbound I-74 segment plotted following this schema.

#### 3.1.2. Results

**Figure 15:**
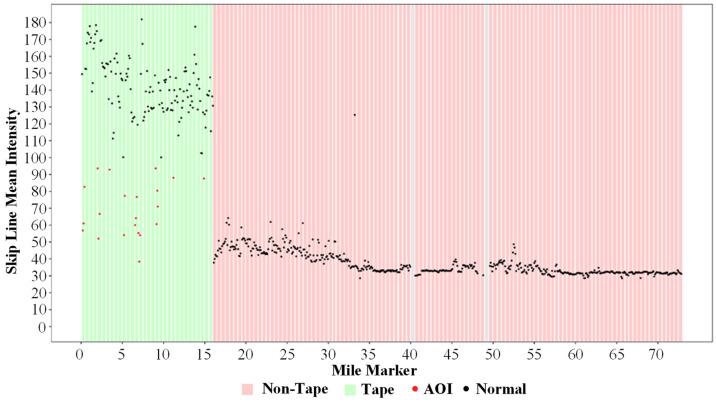
Segmented I-74 LiDAR intensity for skip lines with AOIs automatically identified.

Looking at Figure 14, there is a substantial change in intensity around mile marker 16. This change is caused by the marking type switching from waterborne paint to the much more reflective recessed durable preformed tape. This increase in intensity allows for the segmentation of markings per 0.5-mile bin. The average intensity across the 0.5-mile segments is calculated and subsequently compared to the neighboring segments and a pre-defined intensity threshold of 80 (0–255 Scale). This allows for a very accurate statistical separation of tape and non-tape markings. Once separated, it is possible to generate an inventory of the durable preformed tape markings for any route, as seen in Figure 15. It is also possible to identify individual 0.1-mile segments within the 0.5-mile bins classified as being tape markings that are below an agency-defined intensity threshold and tabulate these areas of interest. For this case, the threshold is set to an arbitrary value of 100 as a proof of concept.

**Figure 16:**
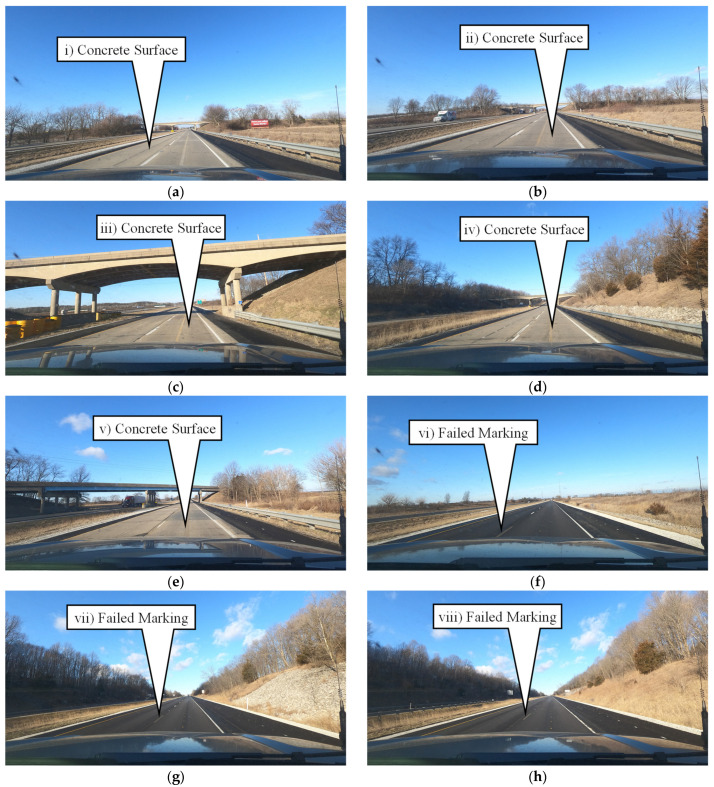
Dashcam images of areas of interest along I-74. (**a**) I-74 W, MM 0.4; (**b**) I-74 W, MM 2.3; (**c**) I-74 W, MM 5.3; (**d**) I-74 W, MM 7.1; (**e**) I-74 W, MM 9.2; (**f**) I-74 W, MM 12.3; (**g**) I-74 W, MM 14.5; (**h**) I-74 W, MM 14.6.

It is also apparent that some areas have a lower intensity compared to the rest of the data in the section of I-74 with the durable preformed markings. Some of these data points have been highlighted by callouts i through viii in Figure 14. Each of these callouts was assessed using a corresponding dashcam image. These dashcam images are shown in Figure 16. Callouts i through v correspond to an area of I-74 where the road surface transitions from hot mix asphalt (HMA) to portland cement concrete (PCC). This change in pavement material is due to a bridge deck above the interstate. The markings in this area were upgraded to the same tape as in the other HMA sections, but there are old waterborne paint markings that were not fully removed as well. This combination of waterborne paint and preformed tape significantly reduces the average intensity of the 0.1-mile segment. Callouts vi through viii correspond to areas where the preformed tape markings need maintenance and therefore cause a lower average intensity for the 0.1-mile segment that they are in. Identifying these outliers is extremely important for agencies to be able to avoid painting over the existing tape markings.

**Figure 17:**
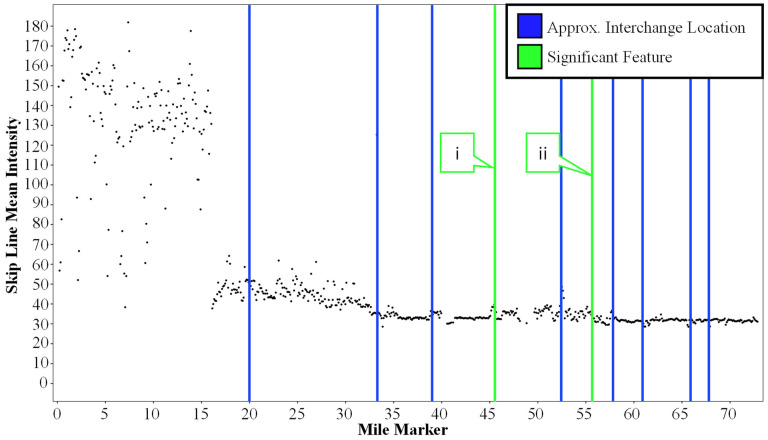
Annotated I-74 LiDAR intensity for skip lines over the 73-mile test segment showing interchange locations and significant features.

Looking at the data for the markings between mile markers 16 and 73, there are also some outliers that have different intensity patterns than the surrounding markings. These differences can be traced back to several sources, including interchanges and significant features. Figure 17 shows an annotated version of the I-74 LiDAR intensity profile with blue and green lines indicating the location of these changes. Callout I points to a PCC underpass and callout ii points to a rest area. Both significant features cause a fluctuation in the intensity profile’s otherwise consistent nature.

### 3.2. West Lafayette Test Loop Case Study

#### 3.2.1. Methods

**Figure 18:**
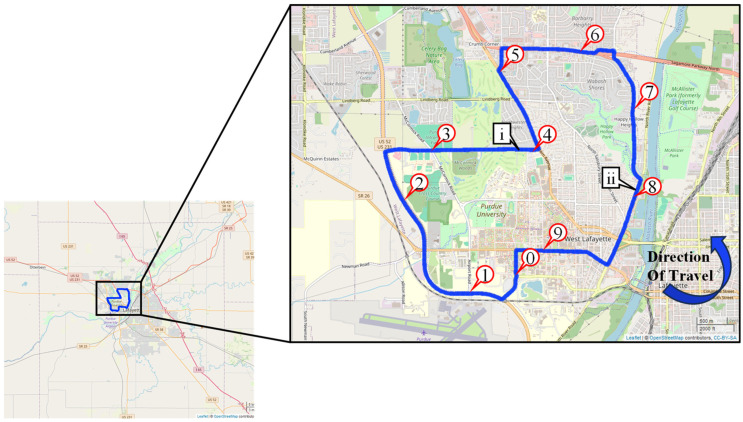
Ten-mile test loop in West Lafayette, Indiana.

After analyzing the data for the high-speed rural interstate segment, a smaller-scale urban data collection was conducted. A 10-mile test segment was selected in West Lafayette, IN, for its diversity of existing markings and the ability to closely examine the lane markings in a lower-speed urban environment. A map of this test loop can be seen in Figure 18. The data collection consisted of six total passes, three traveling clockwise, and three traveling counterclockwise. For consistency, the data were separated into directional groups and processed individually to avoid any cross-contamination. Once the pavement markings were extracted from the point cloud, there were over 600,000 data points, averaging around 100,000 data points per run through the loop. The test loop contains waterborne paint markings, newly installed thermoplastic markings with glass beads, and temporary preformed tape. This tape is similar to the tape used on I-74 but is designed to have less adhesive on it to be used for temporary work zones. These temporary markings also have a lower concentration of reflective glass beads and, therefore, have an overall lower average intensity.

**Figure 19:**
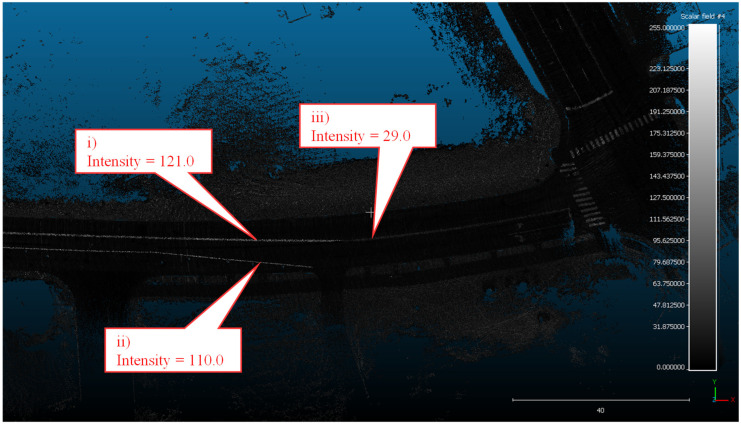
Top-down view of LiDAR-generated point cloud along Cherry Ln with callouts to 3 key locations.

Following the same segmentation algorithm as the I-74 case study, it is possible to identify the location of the temporary preformed tape markings along this test loop. Once the data were collected, it was separated into loops 1–3 for both the clockwise and counterclockwise directions. A sample point cloud can be visualized for a section of Cherry Lane along this test loop in Figure 19. This section of Cherry Ln, near the intersection with Northwestern Ave, marks the beginning of the temporary preformed tape markings installed along a work zone. These markings have a much greater intensity than the existing waterborne paint markings, as seen by the difference in intensity between callouts I and ii and callout iii.

**Figure 20:**
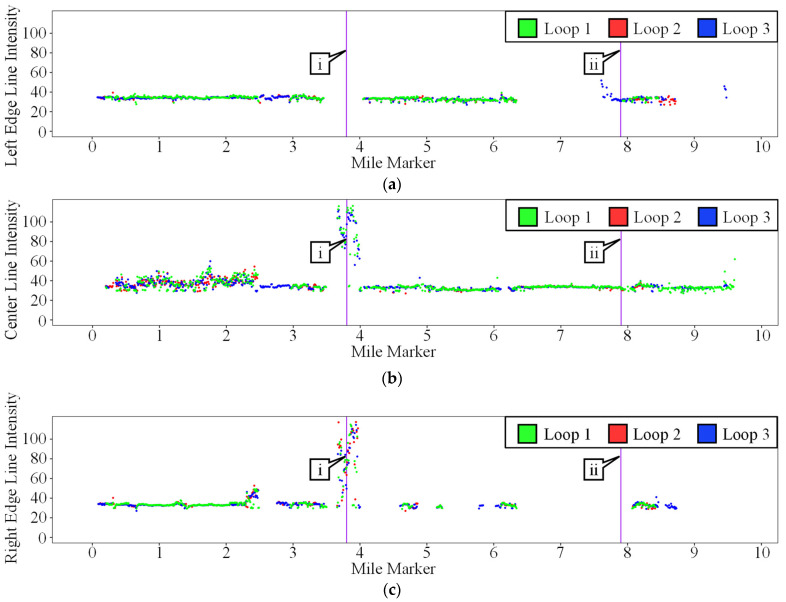
Test loop areas of interest LiDAR intensity profiles. (**a**) Left edge line; (**b**) center line; (**c**) right edge line.

Separate intensity profiles were plotted for the left edge line, centerline, and right edge line. These three plots were stacked vertically and can be seen in Figure 20. Each plot consists of two callouts that point to a purple line behind the data, highlighting two key areas that contain the temporary preformed tape and new thermoplastic markings.

#### 3.2.2. Results

**Figure 21:**
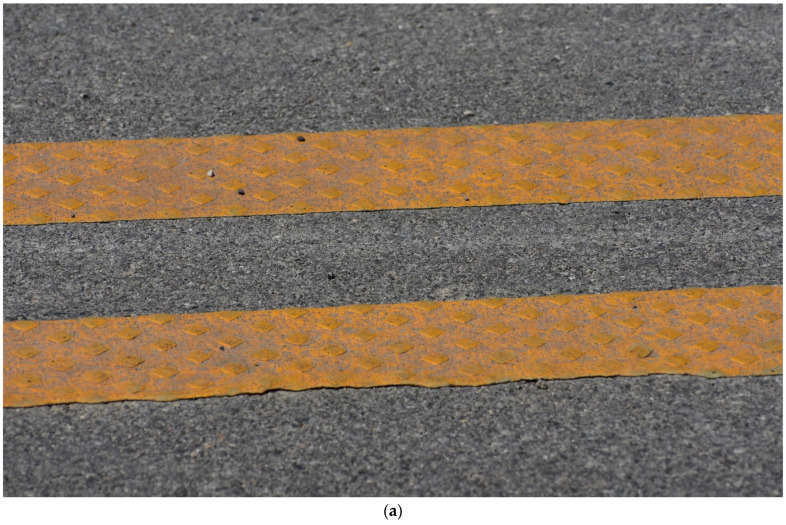

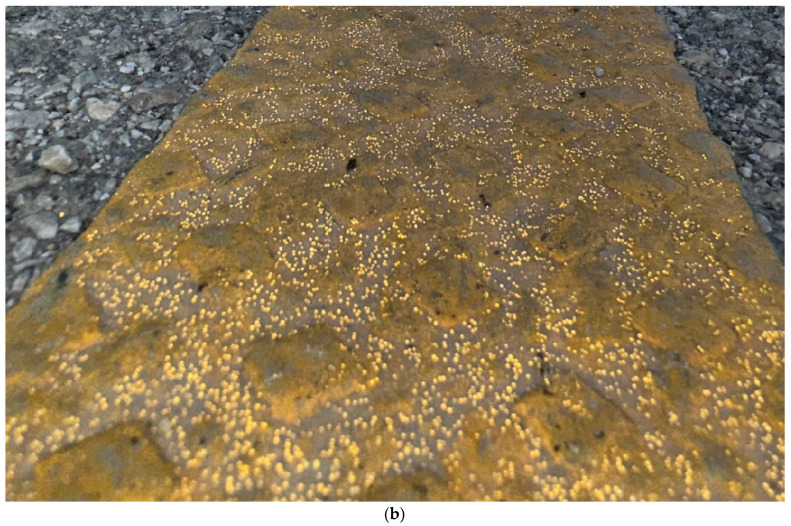
Close-up images of test loop MM 3.9 temporary preformed thermoplastic tape. (**a**) Flash off; (**b**) flash on.

Callout I in Figure 20 corresponds to the area of the test loop along Cherry Lane that contains the temporary preformed tape markings. Figure 20a does not have any data in this section due to the absence of a left edge line along this section of Cherry Lane. Figure 21 contains close-up images of these markings; Figure 21a shows the marking without the camera flash, and Figure 21b shows the marking with the camera flash turned on. These markings have a much higher intensity value than all the other markings along this test loop. This trend is very similar to that of I-74; however, the overall average intensity of these markings is slightly lower. This slightly lower intensity can be explained by the difference between permanent and temporary preformed tape markings as well as dirt and debris located in the work zone, which may be partially covering the markings. The intensity values in Figure 20c near callout I have greater variation than the corresponding values in Figure 20b. This difference is caused by a combination of dirt and construction equipment completely blocking parts of the edge line. 

**Figure 22:**
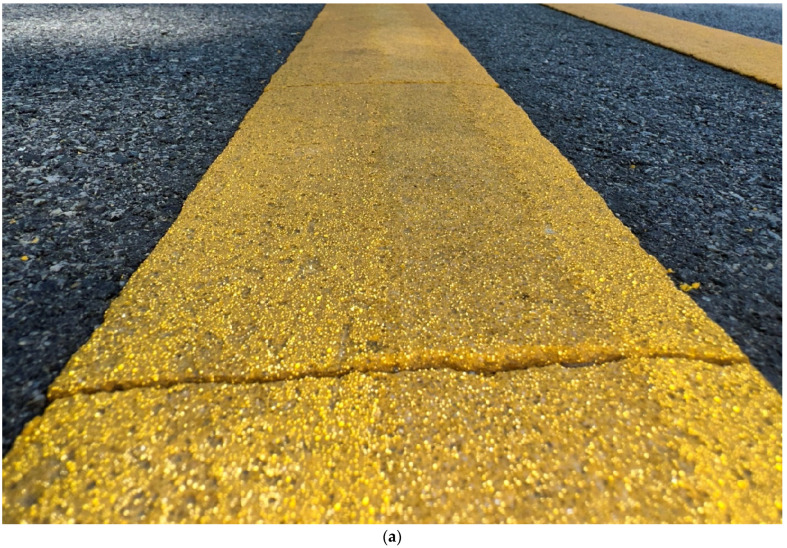

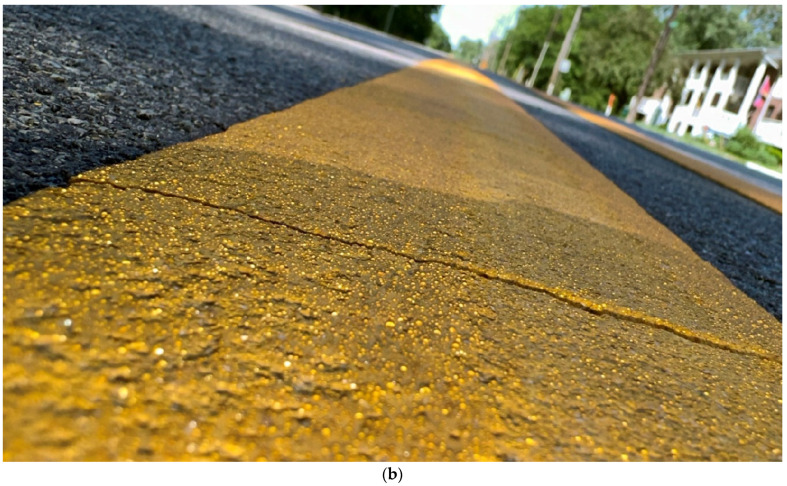
Close-up images of test loop MM 7.9 new thermoplastic markings. (**a**) Overhead view; (**b**) level view.

Callout ii in Figure 20 corresponds to a section along River Road that has newly installed thermoplastic markings with glass beads. These markings can be seen in Figure 22. Figure 22a shows a close-up view of the thermoplastic markings and Figure 22b shows the same marking, but with the camera flash turned on. In theory, these glass beads should be doing the same job as the glass beads in the preformed tape markings, but due to an installation error, the beads are not embedded deep enough inside the thermoplastic material and do not return the light to their source as intended. The top edge of this marking is observed to have been scraped off, presumably by a snowplow. This loss of material is destructive to its retroreflective nature and usually prevented by placing markings in a recessed groove.

## 4. Discussion

### Statewide Scalability and Future Scope

The utilization of high-speed mobile LiDAR-mapping vehicles is a cost-effective and time-friendly approach for statewide pavement marking data collection. The data collected have the potential to be applied to many other research fields, such as ditch line mapping [2], bridge deck analysis [3], pavement quality assessment [4,5], pothole mapping [6], signage visibility [7], etc., making the data highly valuable and robust, reducing the cost per individual data set that gets extracted. Once a statewide database is established, simple algorithms can be applied to identify areas where tape markings exist and indicate areas where they are failing. The filtered data can be programmed into paint trucks to ensure the highly valuable preformed tape markings do not get unintentionally painted over. With machine learning and artificial intelligence becoming readily available, a combination of LiDAR intensity and RGB image data would allow for the automatic identification of marking type, maintenance needs, and even potential remediation strategies.

Repeated data collection over the same marking sections can also be conducted to track the degradation of marking materials over time. Every winter season, many snowplows scrape over the markings, removing the top surface of the material. With most reflective glass beads located on the top surface of the markings, their intensity is diminished by winter maintenance activities. An inventory of historical intensity values can help agencies determine the expected lifecycles of the markings and track which areas are more impacted by winter weather maintenance than others.

## 5. Conclusions

Durable tape and epoxy markings are becoming increasingly common but are installed as part of pavement contracts. This results in a patchwork of pavement markings that are difficult for an agency to maintain an accurate inventory of. With the cost of the tape markings over 20 times more expensive than waterborne paint, it is important that the existing tape not be painted over. Once painted over, the tape loses nearly all its retroreflective properties and the agency effectively loses all its investments (Figure 9).

This study demonstrated the application and scalability of using LiDAR intensity data to classify preformed thermoplastic tape markings from other styles and identify key areas of interest with lower average intensities. Mobile LiDAR-mapping vehicles were deployed along two different test segments. The high-speed rural interstate study covered approximately 73 miles and the low-speed urban study covered approximately 10 miles. The data were segmented into 0.1-mile segments, which allows for easy classification between tape and non-tape markings. Outliers can also be identified and classified to determine areas where the tape needs maintenance and where normal markings have been used. Several RGB images are cross-referenced to the LiDAR intensity data to demonstrate the robustness of LiDAR in classifying pavement markings.

## Figures and Tables

**Table 1:** Indiana Department of Transportation statistics for interstate pavement markings. (a) Cost per lane mile of installed 6” lane markings (thermoplastic, epoxy, and preformed tape include the cost of milling recessed grooves); (b) number of lane miles of recessed pavement markings.

(a)							
	Paint		Thermoplastic		Epoxy		Preformed Tape
	$/mile		$/mile		$/mile		$/mile
**White**	$1425		$7500		$8700		$30,150
**Yellow**	$1525		$8175		$8400		$39,650
**(b)**							
		**Thermoplastic**		**Epoxy**		**Preformed Tape**	
		Number of Lane Miles		Number of Lane Miles		Number of Lane Miles	
**Durable Markings**		1900		2000		800	

**Table 2:** INDOT minimum retroreflectivity values for paint and durable markings.

Material Type	Minimum Initial Retroreflectivity Values $(mcd/m^{2}/lx$)		Retained Values $(mcd/m^{2}/lx$)		
	White	Yellow	Year	White	Yellow
Waterborne Paint	≥250	≥175	1	N/A	N/A
			2	N/A	N/A
Required Minimum	150–249	125–174			
Thermoplastic	≥300	≥200	1	225	150
			2	175	125
Required Minimum	250–299	150–199			
Epoxy (Multi-component)	≥300	≥200	1	225	150
			2	175	125
Required Minimum	250–299	150–199			
Preformed Tape	≥650	≥450	1	400	300
			2	300	200
Required Minimum	550–649	350–449			

**Table 3:** Regression model summary for power and linear models.

	Coefficients		Mean ± STD (RMSE) $(mcd/m^{2}/lx$)	$R^{2}$	r
	a	b			
**Power**$\;(y=ax^{b}$)	1.26	1.69	20 ± 241 (242)	0.76	0.86
**Linear** (y=ax+b)	1.08	−0.13	45 ± 255 (259)	0.74	0.86

## Data Availability

The datasets presented in this article are not readily available because they are part of an ongoing study as well as too large to place in data repositories. Requests to access the datasets should be directed to darcy@purdue.edu.

## References

[1] MUTCD 11th Edition—FHWA MUTCD. https://mutcd.fhwa.dot.gov/kno_11th_Edition.htm.

[2] Lin Y.-C., Manish R., Bullock D., Habib A. (2021). Comparative Analysis of Different Mobile LiDAR Mapping Systems for Ditch Line Characterization. Remote Sens..

[3] Lin Y.-C., Habib A. (2022). Semantic Segmentation of Bridge Components and Road Infrastructure from Mobile LiDAR Data. ISPRS Open J. Photogramm. Remote Sens..

[4] Ravi R., Bullock D., Habib A. (2020). Highway and airport runway pavement inspection using mobile lidar. Int. Arch. Photogramm. Remote Sens. Spat. Inf. Sci..

[5] Ravi R., Bullock D., Habib A. (2021). Pavement Distress and Debris Detection Using a Mobile Mapping System with 2D Profiler LiDAR. Transp. Res. Rec..

[6] Ravi R., Habib A., Bullock D. (2020). Pothole Mapping and Patching Quantity Estimates Using LiDAR-Based Mobile Mapping Systems. Transp. Res. Rec..

[7] Olsen M.J., Parrish C., Che E., Jung J., Greenwood J. (2018). Lidar for Maintenance of Pavement Reflective Markings and Retroreflective Signs.

[8] Hou Q., Ai C., Boudreau N. (2024). An Automated Pavement Marking Retroreflectivity Condition Assessment Method Using Mobile LiDAR and Video Log Images. J. Infrastruct. Syst..

[9] Gao Y., Zhong R., Tang T., Wang L., Liu X. (2017). Automatic Extraction of Pavement Markings on Streets from Point Cloud Data of Mobile LiDAR. Meas. Sci. Technol..

[10] Jung J., Che E., Olsen M.J., Parrish C. (2019). Efficient and Robust Lane Marking Extraction from Mobile Lidar Point Clouds. ISPRS J. Photogramm. Remote Sens..

[11] Mahlberg J.A., Li H., Cheng Y.-T., Habib A., Bullock D.M. (2022). Measuring Roadway Lane Widths Using Connected Vehicle Sensor Data. Sensors.

[12] Mobile LiDAR Deployment Optimization: Towards Application for Pavement Marking Stained and Worn Detection. https://ieeexplore.ieee.org/abstract/document/9672116.

[13] Che E., Olsen M.J., Parrish C.E., Jung J. (2019). Pavement Marking Retroreflectivity Estimation and Evaluation Using Mobile Lidar Data. Photogramm. Eng. Remote Sens..

[14] Zhu J., Bu T., Ma T., Huang X., Chen F. (2024). Raster-Based Point Cloud Mapping of Defective Road Marking: Toward Automated Road Inspection via Airborne LiDAR. J. Transp. Eng. Part B Pavements.

[15] Zhao Y., Burghardt T.E., Li H., Rosenberger P., Babić D., Babić D., Wiesinger F., Helmreich B., Eichberger A. (2024). Enhancing LiDAR Reliability Through Utilization of Premium Road Marking Materials. IEEE Sens. J..

[16] Wang S.-Y., Meng J., Wishart J., Zhao J. (2024). Simultaneous Localization and Mapping with Road Markings Identified from LiDAR Intensity. https://www.researchgate.net/publication/379431452.

[17] Shaon M.R., Orlova E., Jackson E. (2024). Automated Vehicle and Pavement Marking Evaluation in Connecticut.

[18] Katzorke N., Langwaldt L.-M., Schunggart L. (2024). Temporary Road Marking Paint for Vehicle Perception Tests. Appl. Sci..

[19] Jung J., Che E., Olsen M.J., Parrish C.E., Turkan Y., Yoo S. (2024). Instance-Based Clustering of Road Markings with Wear and Occlusion from Mobile Lidar Data. J. Comput. Civ. Eng..

[20] Huston R.G., Wilhelm J.P. (2024). Enhance Road Detection Data Processing of LiDAR Point Clouds to Specifically Identify Unmarked Gravel Rural Roads. J. Auton. Veh. Syst..

[21] Li X., Shang Y., Hua B., Yu R., He Y. (2023). LiDAR Intensity Correction for Road Marking Detection. Opt. Lasers Eng..

[22] Park B.-K.D., Sayer J.R., Clover A.D., Reed M.P. (2023). Longitudinal Degradation of Pavement Marking Detectability for Mobile LiDAR Sensing Technology in Real-World Use. Sensors.

[23] Burghardt T.E., Chistov O., Reiter T., Popp R., Helmreich B., Wiesinger F. (2023). Visibility of Flat Line and Structured Road Markings for Machine Vision. Case Stud. Constr. Mater..

[24] Sauter G., Doring M., Nuyttens R. (2021). High Performance Pavement Markings Enhancing Camera And LiDAR Detection. IOP Conf. Ser. Mater. Sci. Eng..

[25] Yang R., Li Q., Tan J., Li S., Chen X. (2020). Accurate Road Marking Detection from Noisy Point Clouds Acquired by Low-Cost Mobile LiDAR Systems. ISPRS Int. J. Geo-Inf..

[26] Zeybek M. (2021). Extraction of Road Lane Markings from Mobile LiDAR Data. Transp. Res. Rec..

[27] Mahlberg J.A., Cheng Y.-T., Bullock D.M., Habib A. (2021). Leveraging LiDAR Intensity to Evaluate Roadway Pavement Markings. Future Transp..

[28] Cheng Y.-T., Patel A., Wen C., Bullock D., Habib A. (2020). Intensity Thresholding and Deep Learning Based Lane Marking Extraction and Lane Width Estimation from Mobile Light Detection and Ranging (LiDAR) Point Clouds. Remote Sens..

[29] HDL-32E Datasheet.Pdf. https://epan-utbm.github.io/utbm_robocar_dataset/docs/HDL-32E%20datasheet.pdf.

[30] VLP-16-Puck.Pdf. https://www.amtechs.co.jp/product/VLP-16-Puck.pdf.

[31] (2019). Evaluating the Accuracy of Mobile LiDAR for Mapping Airfield Infrastructure—Yi Chun Lin, Yi-Ting Cheng, Yun-Jou Lin, John Evan Flatt, Ayman Habib, Darcy Bullock. https://journals.sagepub.com/doi/full/10.1177/0361198119835802.

[32] Laserlux® G7 Mobile Retroreflectometer. https://www.roadvista.com/products/laserlux-g7-mobile-retroreflectometer.

[33] Cheng Y.-T., Lin Y.-C., Habib A. (2022). Generalized LiDAR Intensity Normalization and Its Positive Impact on Geometric and Learning-Based Lane Marking Detection. Remote Sens..

[34] Retroreflectivity Measurement with RetroTek-D Retroreflectometer. https://retrotekusa.com/retroreflectivity-measurement-retrotek-d-retroreflectometer/.

